# How current law and policy supports providers of NHS healthcare in England to respond to patient harm: A scoping review protocol

**DOI:** 10.1371/journal.pone.0299121

**Published:** 2024-03-11

**Authors:** Naomi Assame, Susan Greenhalgh, John Tingle, Julie Wright, Gillian Yeowell

**Affiliations:** 1 Department of Health Professions, Manchester Metropolitan University, Manchester, United Kingdom; 2 Birmingham Law School, University of Birmingham, Birmingham, United Kingdom; University of Siena: Universita degli Studi di Siena, ITALY

## Abstract

**Introduction:**

Harm arising from National Health Service (NHS) healthcare results in significant human cost for the patient, those who care for them, and the medical staff involved. Furthermore, patient harm results in substantial financial costs to the public purse. Improving how NHS providers in England respond to patient harm could reduce the number of claims for clinical negligence brought against NHS. Doing so will ensure those affected receive the justice they deserve and protect the public purse. Law and policy are key to supporting providers of NHS healthcare to respond to patient harm but are not necessarily understood and therefore can be challenging to apply to practice. Research exploring how law and policy supports this understanding is limited. The purpose of this scoping review is to address this knowledge gap and improve understanding by critically evaluating how law and policy supports providers of NHS healthcare in England to respond to patient harm.

**Methods and analysis:**

The review will use the methodological framework proposed by Arskey and O’Malley, Levac et al and the Joanna Briggs Institute. Search strategies will be developed using selected key words and index terms. MEDLINE, CINAHL, and Westlaw and reference lists of relevant publications will be searched to identify relevant grey literature. Two reviewers will independently assess the extracted data against the eligibility criteria. All studies identified will be charted and the results presented as a narrative synthesis.

## Introduction

Globally, around one in 20 patients are exposed to harm in medical care and can be caused by the actions of healthcare professionals (errors of commission or omission), healthcare system failures or involve a combination of errors made by individuals, system failures and patient characteristics [1]. It is estimated that improving how the National Health Service (NHS) responds to harm could save 928 lives per year and release £98.5 million more for care annually [2]. Reducing the risk of patient harm associated with the delivery of NHS healthcare is a policy priority that is linked to the UK government’s desire to drive down costs associated with claims for clinical negligence brought against the NHS in England.

The annual cost of clinical negligence to the NHS in England currently stands at £13.3 billion [3]. In 2021, the UK Parliament launched the NHS litigation reform inquiry to examine the case for the reform of NHS litigation against a background of a significant increase in costs, and concerns that the clinical negligence process fails to do enough to encourage learning and promote improvements to patient safety.

The National Audit Office, the United Kingdom’s independent spending watchdog, estimate that only 4% of people experiencing harm will make a claim [4]. NHS Resolution is an arm’s length body of the Department of Health and Social Care who indemnify the NHS in England. In 2018, they commissioned and published research that identified that those pursuing a claim for clinical negligence will do so out of frustration because of the absence of a transparent investigation and apology from the healthcare provider of concern [5]. This is despite the presence of law and policy articulating the legal, ethical and moral requirement to be open, honest, and transparent when harm has occurred including providing an apology.

The research findings of NHS Resolution and observations of the National Audit Office suggest that improvements in how NHS providers respond to harm could reduce the number of claims for clinical negligence brought against NHS [4, 5]. Law and policy are key to supporting providers of NHS healthcare to improve their response to patient harm but are not necessarily understood therefore challenging to apply to practice. Greater understanding will improve response to patient harm and ensure those affected receive the justice they deserve as well as protect the public purse. However, there is a paucity of research exploring how law and policy underpins response to harm thus limiting understanding.

## Objective

The objective of this scoping review is to address this knowledge gap by critically evaluating how law and policy supports providers of NHS healthcare in England to respond to patient harm. Completion of this scoping review is crucial to improving understanding and driving the improvements required.

## Methods and analysis

Scoping reviews differ from systematic reviews in that they do not aim to assess the validity and quality of studies to synthesise best practice guidelines. The scoping review methodology aims to map the concepts and evidence available in a particular research area to get a broader understanding of a specific subject [6].

The scoping review will use the Arskey and O’Malley six-stage framework for conducting scoping reviews [6] and also take into consideration the recommendations made by other authors such as Levac [7] and the Joanna Briggs Institute (JBI) manual for evidence synthesis [8]. The JBI Manual for Evidence Synthesis [8] stipulates that a *priori* protocol must be developed before undertaking a scoping review. This scoping review protocol has been drawn up to fulfil this stipulation. A scoping review protocol is important, as it pre-defines the objectives, methods, and reporting of the review and allows for transparency of the process [8]. As per the requirements of the PRISMA extension for Scoping Reviews extension checklist [9], this scoping review protocol is registered with the Open Science Framework (OSF) registries (DOI.10.17605/OSF.IO/8HF9C).

### Research question

The research question for this scoping review forms part of the evidence synthesis phase of a broader research question examining factors that influence decisions around pursuing a claim for clinical negligence in England and supporting providers of healthcare to improve their response to patient harm caused by NHS healthcare. The review question is: **How does law and policy support providers of NHS healthcare in England to respond to harm experienced by patients during the course of their treatment and care.**

Combining a broad research question with a clearly articulated scope of inquiry clarifies the focus of the scoping review and supports the development of an effective search strategy [7]. For the purposes of this review, the phrase ‘respond to harm’ refers to the systems and processes associated with incidents, complaints, and claims for clinical negligence. This further clarification of the research question has supported the development of an effective search strategy and aided the inclusion and exclusion criteria of the retrieved literature.

### Search strategy

Relevant published literature will be identified by searching the peer-reviewed databases MEDLINE, Cumulative Index to Nursing and Allied Health Literature (CINAHL) and Westlaw. Boolean operators (AND/OR) will be used to adjust search parameters.

The three-step search recommended by the JBI Manual for Evidence Synthesis [8] will be carried out in this stage. i) Will use broad search terms to interrogate at least two of the electronic databases identified. This initial search will be followed by an analysis of the text words included in the title and abstract of retrieved records, and of the index terms used to describe the articles. ii) Will utilise all identified keywords and index terms to develop a comprehensive search strategy. iii) will examine the reference list of identified reports. The JBI Manual for Evidence Synthesis [8] recommend that a complete search strategy for at least one major database be included as an appendix in the scoping protocol. A complete search strategy for MEDLINE and CINAHL can be found in Table 1. The search will be conducted on the 18^th^ of December 2023. The date range applied to the search strategy will be for academic studies published between the 2000 and 2023. Only academic studies originating from the United Kingdom and published in English will be included. Bibliographic citations of included studies will be searched to identify relevant law and policy.

**Table 1:** Complete MEDLINE and CINAHL search strategy.

	Keywords / search terms
#1	NHS OR “National Health Service” OR healthcare OR “health care”
#2	Apology OR “saying sorry” OR “duty of candour” OR complaint OR investigation OR compensation OR sue OR claim OR litigation OR “clinical negligence claim” OR “alternative dispute resolution” OR mediation OR inquiry OR inquest Or Fault OR “no fault”
#3	Incident OR “serious incident” OR “medical error” OR “never event” OR “avoidable injury” OR “preventable injury” OR “avoidable harm” OR “preventable harm” OR “patient safety incident”
#4	#1 AND #2 AND #3

Grey literature is “information produced on all levels of government, academia, business and industry in electronic and print formats not controlled by commercial publishing” [10]. For this scoping review, the bibliographic citations of included studies will be searched to identify relevant law and policy.

#### Inclusion and exclusion criteria

*Inclusion criteria*.

- Considers how healthcare providers respond to patient harm
- Consider approaches to investigating patient harm
- Consider approaches to the reduction of patient harm
- Refers to statute, policy or case law

*Exclusion criteria*.

- Does not concern the delivery of care by the NHS in England
- Concerns the conduct of a health professional, dishonesty, fitness to practice
- Concerns the breach of data protection laws
- Concerns the measurement or incidence of patient harm
- Concerns the classification of patient harm
- Establishes the types of injury that arise from patient harm
- Concerns the assessment, diagnosis, treatment or prevention of a clinical pathology
- Concerns measuring the prevalence of clinical pathology
- Concerns harm to health care staff

### Study selection

The three-step search strategy will inform the selection of literature, including grey literature, to be included in the scoping review. Guided by the inclusion and exclusion criteria, the title and abstract will be screened independently by one reviewer (NA). The full text of potentially relevant literature will be reviewed where uncertainty around eligibility exists. A second reviewer (GY) will complete the same process by screening the title and abstract of 10% of the articles retrieved and reviewing 10% of the articles selected for full text review. A third reviewer will be consulted should a disagreement arise regarding the inclusion or exclusion of a study (SG).

**Fig 1:**
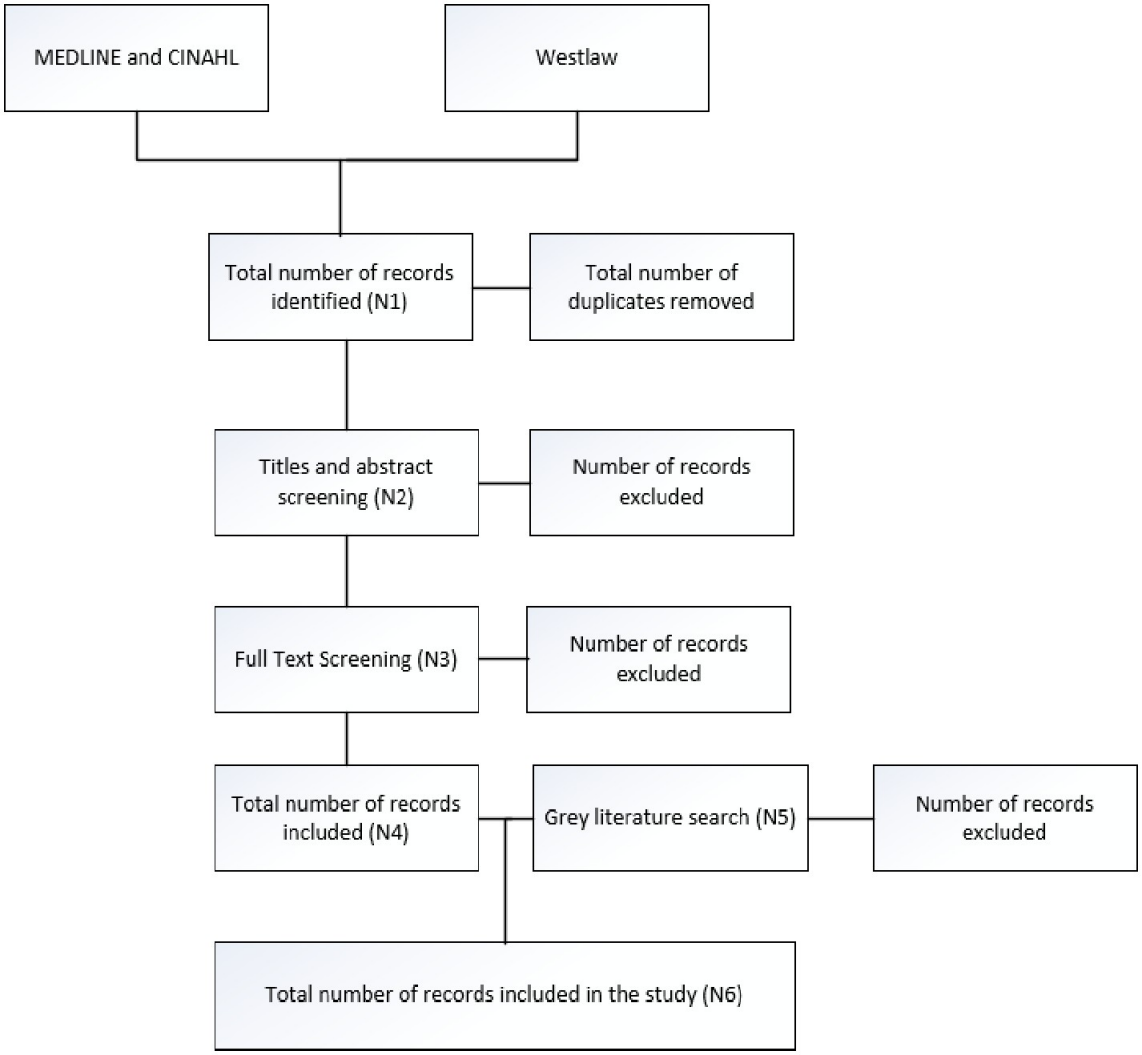
Preferred reporting items for systematic reviews and meta-analyses flow diagram.

The total number of relevant items of literature retrieved from each of the three steps will be recorded. Duplicate studies will be removed, and the number of duplicates removed recorded. The number of studies excluded after screening of titles and abstracts will be recorded along with the reason for exclusion. The number of studies excluded after screening the full text will be recorded with the reason for exclusion. This information will be presented in a Preferred Reporting Items for Systematic reviews and Meta-Analyses (PRISMA) flow diagram, a schematic draft of which is presented in Fig 1 as recommended in the PRISMA extension for Scoping Reviews extension checklist [9]. Results of the search strategy will be managed by utilising RefWorks software. The final search strategy will be fully documented and reported following completion of the scoping review.

### Data extraction

A charting from will be developed using parameters based on those described within the JBI Manual for Evidence Synthesis [8]. Development of the charting form will be an iterative process as the review team continues to extract data, becomes more familiar with the results extracted and updates the form accordingly. To ensure all relevant results are extracted, the charting form will be piloted by different members of the review team and amended as appropriate. The charting form will include the inclusion criteria and explanation of why the study has been included or excluded at this stage in the process. It is anticipated that a similar charting form will be utilised for data identified from the grey literature.

### Data summarization and presentation

Following data extraction, the results will be presented 1) quantitatively i.e., the amount and type of included studies and 2) narratively i.e., a synthesis of all included studies. The implications of the findings to the broader research question examining factors that influence the decision to pursue a claim for clinical negligence will be discussed. Wider discussion will also be made on implications for practice and policy. In addition to being presented as part of doctoral thesis submission, the review will be presented at conferences and published in a peer-reviewed journal.

### Consultation exercise

Stakeholder engagement is crucial to knowledge exchange [7]. For this scoping review, stakeholder engagement will take place at the outset by sharing the protocol with key stakeholders who have expertise in understanding response to patient harm within NHS healthcare. The purpose of sharing the protocol at this phase is to gain the experts opinion on the suggested approach and determine early on whether there are any further considerations that need to be made, particularly for the proposed search strategy.

### Ethics

Ethics approval is not a requirement for this scoping review. All data will be obtained from publicly available documents and, and no primary data will be generated. The research question for this scoping review forms part of the evidence synthesis phase of a broader research question examining factors that influence decisions around pursuing a claim for clinical in England and supporting providers of healthcare to improve their response to patient harm caused by NHS healthcare.

## Discussion

The political and judiciary landscapes constantly change therefore law and policy continuously evolves. A potential limitation of the scoping review is that new law and policy relevant to the research question could be published after the data extraction. It is important to acknowledge this limitation. Regardless, the paucity of research exploring how law and policy underpins response to harm remains thus limiting understanding. We believe that this research is important as it provide policymakers with a greater understanding of how response to patient harm can be improved, ensure those affected receive the justice they deserve as well as protect the public purse.
